# Elemental Phase Partitioning in the γ-γ″ Ni_2_CoFeCrNb_0.15_ High Entropy Alloy

**DOI:** 10.3390/e20120910

**Published:** 2018-11-28

**Authors:** Bin Han, Jie Wei, Feng He, Da Chen, Zhi Jun Wang, Alice Hu, Wenzhong Zhou, Ji Jung Kai

**Affiliations:** 1 Department of Mechanical Engineering, City University of Hong Kong, Hong Kong, China; 2 State Key Laboratory of Solidification Processing, Northwestern Polytechnical University, Xi’an 710072, China; 3 Centre for Advanced Nuclear Safety and Sustainable Development, City University of Hong Kong, Hong Kong, China; 4 City University of Hong Kong, Shenzhen Research Institute, Shenzhen 518057, China

**Keywords:** high entropy alloy, gamma double prime nanoparticles, elemental partitioning, atom probe tomography, first-principles calculations

## Abstract

The partitioning of the alloying elements into the γ″ nanoparticles in a Ni_2_CoFeCrNb_0.15_ high entropy alloy was studied by the combination of atom probe tomography and first-principles calculations. The atom probe tomography results show that the Co, Fe, and Cr atoms incorporated into the Ni_3_Nb-type γ″ nanoparticles but their partitioning behaviors are significantly different. The Co element is much easier to partition into the γ″ nanoparticles than Fe and Cr elements. The first-principles calculations demonstrated that the different partitioning behaviors of Co, Fe and Cr elements into the γ″ nanoparticles resulted from the differences of their specific chemical potentials and bonding states in the γ″ phase.

## 1. Introduction

Recently, a new class of structural materials, known as high entropy alloys (HEAs), have attracted considerable attention due to their excellent properties and potential applications in the aerospace and energy industries [1,2,3,4,5,6,7,8,9,10,11]. Compared with the conventional alloys, the face-centered cubic (FCC) HEAs exhibit unique properties such as outstanding ductility [7], exceptional fracture toughness [12] as well as excellent corrosion resistance [13]. However, the single-phase FCC HEAs are insufficiently strong, which limits their engineering applications.

The strategy of introducing the dispersed hard D0_22_-structured gamma double prime (γ″) or L1_2_-structured gamma prime (γ′) nanoparticles into the FCC matrix (γ phase) has been proved to be one of the most effective approaches to enhance the strength of the FCC HEAs, as it is the case in many superalloys [10,14,15,16]. It is known that the alloying elements in the γ″ or γ′ phase plays an important role on the stability and the mechanical properties of the nano-precipitated alloys [17,18,19,20]. Therefore, it is critical to clarify the partitioning of the alloying elements into the nanoparticles of the FCC HEAs. However, this issue still lacks research because the observation of alloying elements in the nanoparticles which embedded in the FCC matrix is still a challenge. Although the energy dispersive X-ray spectroscopy (EDS) equipped on scan electron microscope (SEM) or on transmission electron microscope (TEM) has been widely used to determine the material composition, it is difficult to distinguish the composition of the nanoparticles from that of the surrounding matrix. Atom probe tomography (APT), the only technique which can generate the three-dimensional (3D) atom maps of materials in the real space with nearly atomic-scale resolution, has been proved to be a powerful method of characterizing the composition of different kinds of nanoparticles [15,18,21,22]. Recently, we have successfully clarified the partitioning of the alloying elements into the γ′ nanoparticles in the NiFeCoCrTi_0.2_ HEA by APT [22]. In this work, the γ″ nanoparticles in a Ni_2_CoFeCrNb_0.15_ HEA was investigated by APT. Different partitioning behaviors of the alloying elements into the γ″ nanoparticles were observed. It was found that the Co element tends to partition into the γ″ nanoparticles but Fe and Cr elements are largely depleted from the γ″ nanoparticles. The APT results were confirmed by the first-principles calculations from the perspective of the electronic states.

## 2. Materials and Methods

An ingot with a composition Ni_2_CoFeCrNb_0.15_ was produced by arc melting Fe, Co, Ni, Cr, and Nb metals with high purity (>99.9%) in an argon atmosphere. After repeatedly melted five times, the ingot was then drop-casted into a copper mold to make a slab with a dimension of 5 mm × 10 mm × 50 mm. Afterwards, the slab was solution-treated at 1473 K for 2 h, followed by water quenching. Then, the homogenized slab was cold rolled with a total thickness reduction of 70% and subsequently recrystallized at 1473 K for 4 minutes (min) and water-quenched. At last, aging was performed at 923 K for 40 h and 100 h, respectively, followed by water quenching.

The TEM specimen was prepared by mechanically grinding and followed by ion-milling using a precision ion polishing system (PIPS, Model 695, Gatan, Pleasanton, CA, USA). The TEM (TEM, JEOL 2100F, Tokyo, JAPAN) was operated under 200 keV. Needle specimens for APT analysis were prepared by gallium focused-ion-beam (FIB), with a FIB-SEM dual-beam system (Scios, FEI, Hillsboro, OR, USA), using a conventional lift-out technique [23]. The APT analysis was performed using a local electrode atom probe (LEAP5000 XR, CAMECA, Madison, WI, USA). The samples were run in the voltage mode at a specimen temperature of 50 K, with 200 kHz pulses at a pulse fraction of 20%. An Integrated Visualization and Analysis Software (IVAS, Version 3.8.2) protocol was employed to reconstruct the 3D atomic maps [24].

## 3. Results and Discussion

**Figure 1:**
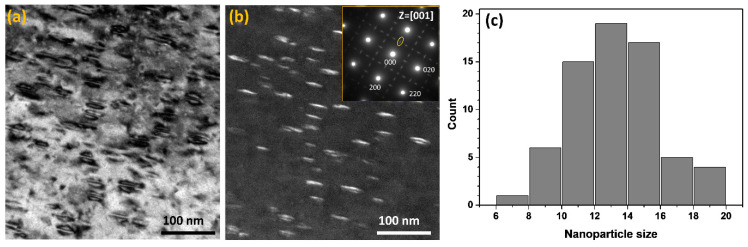
The BF-TEM (**a**); and the DF-TEM (**b**) images of the sample aged for 40 h. The inset in (**b**) is the SADP along the zone-axis z = [001]. (**c**) The size distribution of the γ″ nanoparticles.

To confirm the formation of D0_22_-structured γ″ nanoparticles, the TEM analysis was performed before the APT measurement. Figure 1 shows a bright-field (BF) and a dark-field (DF) TEM images of the sample aged for 40 h. The DF-TEM image recorded from the spot marked with a yellow circle in the inset selected area diffraction pattern (SADP). The nanoparticles can be clearly observed in both the BF-TEM and the DF-TEM images. From the SADP it can be confirmed that the matrix has an FCC structure (γ phase), whilst the nanoparticles have a D0_22_ structure (γ″ phase) which is revealed by the additional faint spots. Figure 1c shows the size distribution of the nanoparticles with an average size of 13.5 ± 2.9 nm. The size of the nanoparticles was measured from the length of the nanoparticle along their long axis.

**Figure 2:**
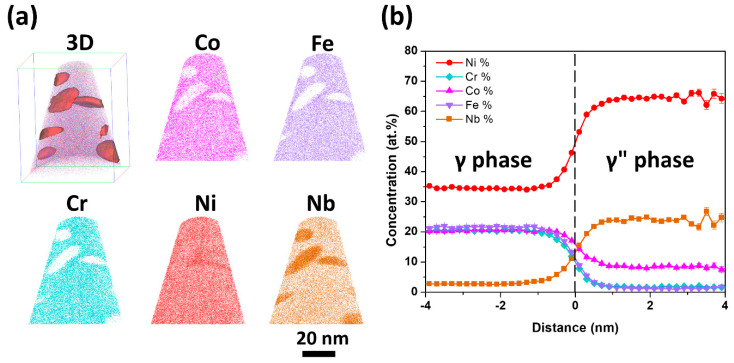
(**a**) The 3D atom map (62 × 64 × 80 nm^3^) and the 4 nm-thick sliced atom maps of Co, Fe, Cr, Ni, and Nb of the 40 h aged sample. In the 3D map, the nanoparticles are delineated by 50 at.% Ni iso-concentration surfaces in red for better illustration. (**b**) The proximity histogram of the iso-concentration surfaces illustrated in the 3D atom map. The alloying elements are shown as a function of the distance from the iso-concentration surface (vertical dashed line).

Figure 2 shows the APT results of the sample aged for 40 h. In the three-dimensional (3D) atom map, the γ″ nanoparticles are delineated by 50 at.% Ni iso-concentration surfaces in red. It can be observed that the γ″ nanoparticles are disk-like. From the sliced atom maps, it can be found that the γ″ nanoparticles mainly consist of Ni and Nb. In addition, Co element shows a strong tendency to partition into the γ″ nanoparticles, but Fe and Cr elements are largely depleted from the γ″ nanoparticles. To clarify the accurate composition of these γ″ nanoparticles, the proximity histogram, which is calculated over the iso-concentration surfaces, is plotted (Figure 2b). Therein, the chemical elements are displayed as a function of the distance from the iso-concentration surfaces. The proximity histogram shows that the average concentration of Co is up to 8.2 ± 0.3 at.%, but the average concentrations of Fe and Cr are only 1.3 ± 0.4 and 1.6 ± 0.1 at.% in the γ″ nanoparticles, respectively. To investigate the composition stability of γ″ nanoparticles, the sample aged for 100 h was also analyzed by APT. The composition of the γ″ nanoparticles are summarized in Table 1. It was found that the composition of γ″ nanoparticles in the sample aged for 100 h is almost the same as that in the sample aged for 40 h, which indicates that the composition of γ″ nanoparticles have reached the steady state after 40 h aging. The APT results demonstrate that the Co element is much easier to partition into the γ″ nanoparticles than Fe and Cr elements. In addition, Co, Fe, and Cr prefer Ni sublattice sites in the Ni_3_Nb-type γ″ nanoparticles because the Ni composition in the γ″ nanoparticles are only 65 at.%. It should be noted that a small part of Co, Fe, or Cr atoms may also occupy Nb sublattice sites as the Nb composition in the γ″ nanoparticles is about 24%. However, it is difficult to determine which kind of elements occupied Nb sublattice sites only from APT results. Some similar results are also found in the Ni-based superalloys [25,26,27]. For example, Lawitzki et al. reported that the alloying elements such as Cr occupied both the Ni and Nb sublattice sites of γ″ phase in the 718 alloy [25].

First-principles calculations were performed to confirm the site substitution preferences in the γ″ nanoparticles and to investigate the origin of the different partitioning behaviors of the Co, Fe, and Cr elements into the γ″ nanoparticles. The calculations employed the plane-wave pseudopotential approximations with the generalized gradient approximations, as implemented in the Vienna ab initio simulation package (VASP) [28]. A plane wave cutoff energy of 500 eV and 9 × 9 × 9 Monkhorst–Pack k-point grids were used in the calculation. A 3D periodic supercell with D0_22_-structured Ni_24_Nb_8_ was employed to determine the total energies of the cells. The D0_22_-structured Ni_3_Nb was fully relaxed, and the lattice parameters were determined to be a = b = 3.643 Å and c = 7.484 Å, which is in good agreement with both the previous reported experimental and theoretical results [27,29].

The formation energies for an element X (X = Co, Fe, and Cr) to substitute a Ni site and a Nb site of the D0_22_-structured Ni_3_Nb were defined as [30]

(1) $$E_{X\to Ni}=\left(E_{Ni_{23}XNb_{8}}^{tot}+\mu_{Ni}\right)-\left(E_{Ni_{24}Nb_{8}}^{tot}+\mu_{X}\right)$$

(2) $$E_{X\to Nb}=\left(E_{Ni_{24}Nb_{7}X}^{tot}+\mu_{Nb}\right)-\left(E_{Ni_{24}Nb_{8}}^{tot}+\mu_{X}\right)$$

where *E^tot^* is the total energy and *μ* is the chemical potential. The chemical potential is defined as the energy per atom of the element in its stable pure phase. Our calculations show that the total energy of *Ni*_24_*Nb*_8_ ($E_{Ni_{24}Nb_{8}}^{tot}$) is −222.84 eV and the chemical potentials of Ni and Nb are −5.47 and −10.20 eV, respectively. Table 2 summarized the total energies, the chemical potentials, and the formation energies. The calculation results demonstrate that Co and Fe atoms prefer to occupy the Ni sublattice sites rather than Nb sublattice sites, as the formation energies, *E_Co/Fe_→_Ni_*, are significantly lower than *E_Co/Fe_→_Nb_*. However, the formation energies for Cr to occupy Ni and Nb sublattice sites are almost same, which indicates that Cr atoms occupy both the Ni and Nb sublattice sites. In addition, the formation energy of Co that occupies the Ni sublattice site is nearly zero, which is much lower than that of Fe and Cr, indicating that Co is more stable in the D0_22_-structured Ni_3_Nb than Fe and Cr. Similar results were also reported in the L1_2_-structured Ni_3_Ti phase [31]. The calculation results confirmed the APT observation that the concentration of Co in the γ″ nanoparticles are much higher than that of Fe and Cr.

**Figure 3:**
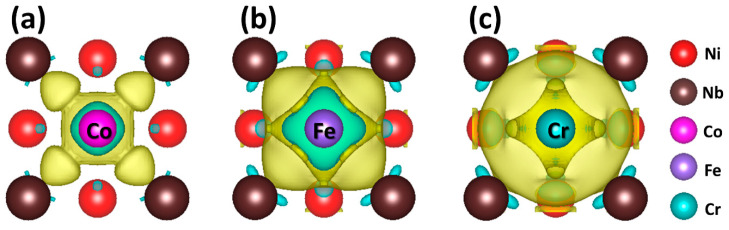
The charge density difference on the (001) plane of the Ni_23_CoNb_8_ (**a**), Ni_23_FeNb_8_ (**b**), and Ni_23_CrNb_8_ (**c**) systems with reference to the Ni_24_Nb_8_ system. The yellow regions and blue regions correspond to the increased and decreased charge density (0.001 eV/Bohr^−3^), respectively.

To further clarify the origin of the formation energy differences of Co, Fe, and Cr in the D0_22_-structured Ni_3_Nb, the total energy, and the chemical potential of the solute atoms are carefully checked, as the formation energy is determined by these two parts. It is found that the formation energy difference between Fe and Cr is mainly caused by the difference of their chemical potentials because the total energies of $E_{Ni_{23}FeNb_{8}}^{tot}$ and $E_{Ni_{23}CrNb_{8}}^{tot}$ are almost the same. However, both the chemical potential and the total energy of Co ($E_{Ni_{23}CoNb_{8}}^{tot}$) are higher than that of Fe and Cr, which indicates that the formation energy differences between Co and Fe/Cr not only result from their chemical potential differences, but also come from their total energy differences. As the total energy originates from the charge distribution of the system, we calculated the charge density difference of Ni_23_CoNb_8_, Ni_23_FeNb_8_, and Ni_23_CrNb_8_ systems with reference to Ni_24_Nb_8_ system, respectively. From the charge density difference, we can find that the charge accumulation appears between Co and Nb atoms (Figure 3a) but does not appear between Fe and Nb (Figure 3b), or Cr and Nb (Figure 3c). The charge distribution results indicate that the bonding for Co and Nb is much stronger than that of Fe and Cr. The strong Co-Nb bond stabilizes Co atoms in the D0_22_-structured Ni_3_Nb, which demonstrates that the bonding state of Co plays an important role in lowering the formation energy compared with that of Fe and Cr.

## 4. Conclusions

In summary, the partitioning of alloying elements into the γ″ nanoparticles in a Ni_2_CoFeCrNb_0.15_ HEA was studied by APT and first-principles calculations. It was found that the composition of Co in the γ″ nanoparticles is up to 8.2 at.% but the composition of Fe and Cr are only 1.3 at.% and 1.6 at.%, respectively. This indicates that the Co element is much easier to partition into the γ″ nanoparticles than Fe and Cr elements. In addition, Co and Fe atoms prefer to occupy the Ni sublattice sites but Cr occupies both Ni and Nb sublattice sites. The first-principles calculations demonstrated that the different partitioning behaviors of Co, Fe, and Cr elements into the γ″ nanoparticles are attributed to the differences of their specific chemical potentials and the bonding states in the γ″ phase. This research paves the way for the composition control of the γ″ nanoparticles in the HEAs.

## Figures and Tables

**Table 1:** Chemical composition of γ″ nanoparticles (at.%).

Aging Time	Co	Fe	Cr	Ni	Nb
40 h	8.2 ± 0.3	1.3 ± 0.1	1.6 ± 0.1	64.3 ± 0.4	24.6 ± 0.2
100 h	8.1 ± 0.1	1.2 ± 0.1	1.4 ± 0.1	65.2 ± 0.3	24.0 ± 0.2

**Table 2:** The Calculated chemical potentials, total energies and formation energies with the unit of eV.

	*μ*	$E_{Ni_{23}XNb_{8}}^{tot}$	$E_{Ni_{24}Nb_{7}X}^{tot}$	$E_{X\to Ni}$	$E_{X\to Nb}$
Co	−7.01	−224.16	−217.74	0.08	1.71
Fe	−8.23	−225.00	−218.79	0.19	1.61
Cr	−9.50	−225.55	220.65	1.38	1.50
